# MicroRNA-126-3p/5p and Aortic Stiffness in Patients with Turner Syndrome

**DOI:** 10.3390/children9081109

**Published:** 2022-07-23

**Authors:** Masood Abu-Halima, Felix Sebastian Oberhoffer, Viktoria Wagner, Mohamed Abd El Rahman, Anna-Maria Jung, Michael Zemlin, Tilman R. Rohrer, Eckart Meese, Hashim Abdul-Khaliq

**Affiliations:** 1 Institute of Human Genetics, Saarland University, 66421 Homburg, Germany; viktoria.wagner@ccb.uni-saarland.de (V.W.); eckart.meese@uks.eu (E.M.); 2 Department of Pediatric Cardiology, Saarland University Hospital, 66421 Homburg, Germany; felix.oberhoffer@med.uni-muenchen.de (F.S.O.); mohamed.abd-el-rahman@uks.eu (M.A.E.R.); hashim.abdul-khaliq@uks.eu (H.A.-K.); 3 Division of Pediatric Cardiology and Intensive Care, University Hospital, LMU Munich, 81377 Munich, Germany; 4 Center for Clinical Bioinformatics, Saarland University, 66123 Saarbruecken, Germany; 5 Department of General Pediatrics and Neonatology, Saarland University Hospital, 66421 Homburg, Germany; anna-maria.jung@uks.eu (A.-M.J.); michael.zemlin@uks.eu (M.Z.); tilman.rohrer@uks.eu (T.R.R.)

**Keywords:** Turner Syndrome, MicroRNAs, vascular dysfunction

## Abstract

Background: Turner Syndrome (TS) is a relatively rare X-chromosomal disease with increased cardiovascular morbidity and mortality. This study aimed to identify whether the circulating miR-126-3p/5p are involved in the pathophysiology of vascular dysfunction in TS. Methods: Using the RT-qPCR, the abundance levels of miR-126-3p and miR-126-5p were determined in 33 TS patients and 33 age-matched healthy volunteers (HVs). Vascular screening, including the assessment of blood pressure, pulse wave velocity, augmentation index, aortic deformation, arterial distensibility, and arterial elastance, was conducted in TS patients and HVs. Results: The abundance levels of miR-126-3p and miR-126-5p were significantly higher in TS patients compared to HVs (*p* < 0.0001). Within the TS cohort, miR-126-3p/5p correlated significantly with aortic deformation (r = 0.47, *p* = 0.01; r = 0.48, *p* < 0.01) and arterial distensibility (r = 0.55, *p* < 0.01; r = 0.48, *p* < 0.01). In addition, a significant negative correlation was demonstrated between miR-126-3p and arterial elastance (r = −0.48, *p* = 0.01). The receiver operating characteristic analysis showed that miR-126-3p and miR-126-5p separated the tested groups with high sensitivity and specificity. Conclusions: The abundance levels of miR-126-3p and miR-126-5p were significantly higher in TS patients compared to HVs. Within the TS cohort, a lower abundance level of miR-126-3p and miR-126-5p was linked with a significantly higher aortic stiffness.

## 1. Introduction

Turner Syndrome (TS), a relatively rare chromosomal disease, is defined by the entire or partial loss of one of the two X chromosomes in females [1]. Roughly 1:2500–3000 female newborns is affected by TS [1]. Compared to the general population, the cardiovascular morbidity and mortality of TS patients are reported to be significantly increased [2]. Up to 50% of TS girls and TS women present with a congenital heart disease, of which the bicuspid aortic valve and the coarctation of the aorta represent the most common forms [3,4]. Moreover, aortic dilatation is frequently seen in this patient group [3]. Numerous studies were able to demonstrate a systemic vascular dysfunction in TS patients, visualized by an elevated arterial stiffness [5,6,7,8]. The systemic vascular dysfunction in TS might be due to the increased prevalence of glucose metabolism disorders, lipid metabolism disorders, excess weight, and arterial hypertension in this cohort, aggravating the process of atherosclerosis [3,9]. In addition, intrinsic factors that lead to an abnormal aortic wall structure and function have been assumed in TS [5]. The combination of the above-mentioned cardiovascular risk factors might explain the increased threat for acute aortic dissection in TS, which is considered to be up to 100-fold higher compared to the general population [10]. MicroRNAs (miR, miRNA) are small, non-coding RNAs, which are involved in the post-transcriptional regulation of gene expression in cellular and biological processes [11,12]. Currently, 2300 “real” miRNAs have been validated in the miRNA database [13]. It is assumed that almost every biological and cellular process is regulated by miRNAs, including processes controlling congenital heart disease [14,15,16,17,18,19,20,21]. Studies have shown that miRNAs are engaged in the pathology of atherosclerosis, arterial stiffening, aortic dilatation, and aortic dissection [22,23,24,25]. In a recent study, we identified circulating miRNA signatures in TS patients, including those TS patients with and without congenital heart disease [17]. Interestingly, miR-126-3p abundance levels were significantly higher in TS patients compared to healthy controls. Within the TS group, the miRNA-126-3p abundance level was significantly lower in subjects displaying congenital aortic valve disease, and correlated significantly with the ascending aortic diameter, suggesting its involvement in the vascular morbidity of TS [17]. In the literature, two strands of miR-126, specifically miR-126-3p and miR-126-5p, have been reported to play an important role in the pathophysiology of atherosclerosis [26,27]. MiR-126-5p, in particular, is considered to limit atherosclerotic lesion formations by promoting endothelial proliferation [28]. This study aimed to identify whether the circulating miR-126-3p and miR-126-5p are potentially involved in the pathophysiology of vascular dysfunction in TS.

## 2. Methods

### 2.1. Ethical Statement

The study was conducted according to the guidelines of the Declaration of Helsinki and was approved by the Ethics Committee of the Ärztekammer des Saarlandes (Saarbruecken, Germany) (Ethical vote No. 07/18, date of approval: 23 March 2018). All participants gave written informed consent before enrollment. In minor study participants, written informed consent was obtained from parents or legal guardians.

### 2.2. Study Population and Blood Collection

A total of 33 TS patients with an approved genetic diagnosis who were treated at the Department of Pediatric Cardiology and/or at the Department of General Pediatrics and Neonatology—Section of Pediatric Endocrinology and Diabetology—of Saarland University Hospital were enrolled. The assessment of karyotype and cardiovascular morbidity in the TS cohort was extensively portrayed in a recent publication of our departments [17]. Thirty-three healthy and age-matched females served as healthy volunteers (HVs). In these subjects, the absence of any cardiovascular condition was verified by a physical examination and echocardiography. The vascular screening was conducted in all TS subjects and in 14 HVs. No significant difference in age between both groups was assessed. From all studied participants, 2.5 mL of venous blood was taken and injected into PAXgene™ blood tubes (Becton–Dickinson, Heidelberg, Germany). PAXgene™ blood tubes were then stored for 24 h at room temperature to assure complete blood cell lysis, followed by a −20 °C storage for multiple days, and finally, they were transferred to −80 °C for long-term storage until RNA and miRNA isolation.

### 2.3. Assessment of Vascular Function

Detailed information on the vascular function assessment has been previously described [6,29,30] and is shortly summarized as follows.

#### 2.3.1. Oscillometric Pulse Wave Analysis

An automatic oscillometric blood pressure measuring device (Mobil-O-Graph, IEM GmbH, Stolberg, Germany) was used to assess the following parameters non-invasively: brachial systolic blood pressure (SBP, mmHg), brachial diastolic blood pressure (DBP, mmHg), brachial pulse pressure (PP, mmHg), central systolic blood pressure (cSBP, mmHg), central diastolic blood pressure (cDBP, mmHg), central pulse pressure (cPP, mmHg), augmentation pressure (mmHg), augmentation index adjusted to a heart rate of 75 bpm (AIx@75, %), and pulse wave velocity (PWV, m/s). Before oscillometric pulse wave analysis, study participants were asked to rest in a seated position for at least 5 to 10 min. Cuff sizes were selected according to left upper arm circumference.

#### 2.3.2. Abdominal Aortic Strain and Arterial Distensibility

A 2.5–3.0-MHz phased array transducer and a Vingmed Vivid 9 ultrasound system (GE Healthcare, Fairfield, CT, USA) were used for sonographic examination. M-Mode of the abdominal aorta was performed in a long subxiphoid view at the epigastric level. Minimal (AAO-D_min_, cm) and maximal (AAO-D_max_, cm) abdominal aortic diameters were assessed offline on a separate workstation (EchoPAC PC version 202, GE Healthcare, Fairfield, CT, USA). Abdominal aortic strain (AAO-S, %), visualizing abdominal aortic deformation, was characterized as

(1) $$\mathrm{AAO}-S=\frac{\mathrm{AAO}-D_{\max}\;-\;\mathrm{AAO}-D_{\min}}{\mathrm{AAO}-D_{\min}}$$

Arterial distensibility (mmHg^−1^ × 10^−3^), a marker of abdominal aortic elasticity, was defined as

(2) $$\mathrm{Arterial}\;\mathrm{Distensibity}=\frac{\left(2\times \;\mathrm{AAO}-S\right)}{\mathrm{SBP}-\mathrm{DBP}}$$

#### 2.3.3. Arterial Elastance

A 2.5–3.5-MHz phased array transducer and a Vingmed Vivid 9 ultrasound system (GE Healthcare, Fairfield, CT, USA) were used for echocardiographic examination. Recorded clips were analyzed offline on a separate workstation (EchoPAC PC version 202, GE Healthcare, Fairfield, CT, USA). Arterial elastance (Ea, mmHg/mL), representing left ventricular afterload, was defined as

(3) $$\mathrm{Ea}=\frac{\mathrm{End}-\mathrm{Systolic}\;\mathrm{Blood}\;\mathrm{Pressure}}{\;\mathrm{Left}\;\mathrm{Ventricular}\;\mathrm{Stroke}\;\mathrm{Volume}}$$

The end-systolic blood pressure (Pes, mmHg) was calculated as:

(4) Pes= SBP ×0.9

Left ventricular stroke volume was assessed through M-Mode echocardiography in parasternal long axis view. Ea was indexed to body surface area (BSA, m^2^).

### 2.4. Preparation of RNA, RNA Quality Control for MicroRNA Analysis

Total RNA, including miRNAs, was purified from blood samples using PAXgene™ Blood miRNA Kit on the QIAcube™ robot (Qiagen, Hilden, Germany), and Dnase I treatment (Qiagen) was performed to remove the residual genomic DNA contamination. The concentration and purity of isolated total RNA were measured using a NanoDrop ND-2000 spectrophotometer (Thermo Fisher Scientific, Waltham, MA, USA). The RNA integrity was assessed using the Agilent Bioanalyser 2100 Eukaryote Total RNA Nano Series II (Agilent Technologies, Santa Clara, CA, USA). All RNA samples passed quality control with an RNA integrity number of ≥7.0.

### 2.5. Analysis of Single miRNAs by RT-qPCR

Using the RT-qPCR, the abundance levels of hsa-miR-126-3p and hsa-miR-126-5p were determined in 33 patients with TS and 33 age-matched HVs using the StepOnePlus™ Real-Time PCR System (Applied Biosystems, Foster City, CA, USA) and the TaqMan^®^ microRNA Assays (Thermo Fisher Scientific), according to the manufacturer’s recommendations. Briefly, complementary DNA (cDNA) was generated in 15-µL reactions by the reverse transcription (RT) of 75 ng total RNA using the TaqMan^®^ MicroRNA Reverse Transcription Kit and RT Primers Pool from the hsa-miR-126-3p (Assay ID: 002228) and hsa-miR-126-5p (Assay ID: 000451), along with the small nuclear RNA (snRNA) RNU6B (Assay ID: 001093). Following the RT step, qPCR was carried out using 1.0 µL of each TaqMan Primer Assay (20X) (a mixture of forward and reverse primers and a probe) (Thermo Fisher Scientific), 2.0 µL of RT reaction (1:5 diluted), 10.0 µL of 2XtaqMan™ Universal PCR Master Mix, no AmpErase™ UNG (Thermo Fisher Scientific), and 7.0 μL of DEPC-treated water to obtain a final volume of 20 μL. All RT-qPCR experiments were carried out in duplicates using the Liquid Handling Robot QIAgility™ (Qiagen) before performing qPCR according to the manufacturer’s recommendations.

### 2.6. Statistical Analysis

GraphPad Prism Software version 7 (GraphPad Software) and SPSS (IBM SPSS Statistics for Windows, version 26.0.) were used for statistical analysis. Prior power analysis with the α and β error probability of 0.05 showed that ≥ 30 samples per group were required for the appropriate miRNA statistical analysis. The Mann–Whitney U test was used to evaluate the differences in miR-126-3p/5p along with RNU6B as an endogenous reference between the patients with TS and HVs. The relative quantitative method of 2^−ΔCq^ was used to measure the dynamic change of miR-126-3p/5p. ROC curves and AUC were established to evaluate the diagnostic value of miR-126-3p/5p to differentiate between TS patients and HVs. Spearman’s regression coefficient and the difference between the groups were analyzed using an unpaired-two-tailed *t*-test. Continuous clinical variables were tested for normal distribution using the Kolmogorov–Smirnov test. Variables were presented as mean ± standard deviation if normally distributed and as median (range) if non-normally distributed. The unpaired Student’s *t*-test was utilized to compare continuous variables with normal distribution. Non-normally distributed continuous variables were compared using the Mann–Whitney U test. MiRNAs and clinical data were considered as differentially abundant if they obtained an adjusted *p*-value of <0.05.

## 3. Results

### 3.1. Clinical Characteristics of Turner Syndrome Patients and Healthy Volunteers

Precise patients’ characteristics of TS subjects, including karyotype as well as cardiovascular morbidity, and of HVs, who underwent vascular screening, have been previously described [17]. TS patients and HVs did not differ significantly in age (17.11 (8.66–44.13) years vs. 17.86 (12.93–43.82) years) and weight (53.05 ± 17.88 kg vs. 57.08 ± 8.26 kg) [17]. However, significant differences in body height (147.52 ± 11.59 cm vs. 165.64 ± 6.64 cm, *p* < 0.001), body mass index (23.90 ± 6.03 kg/m^2^ vs. 20.75 ± 2.41 kg/m^2^, *p* = 0.014), and body surface area (1.46 ± 0.28 m^2^ vs. 1.62 ± 0.14 m^2^, *p* = 0.015) were assessed [17].

### 3.2. Vascular Function

A significantly increased heart rate was demonstrated in the TS group compared to HVs. In addition, AIx@75 tended to be increased in TS patients but did not reach statistical significance. Parameters on vascular function are summarized in Table 1 for both groups.

### 3.3. Abundance Level of miR-126-3p and miR-126-5p in the Blood by RT-qPCR

**Figure 1:**
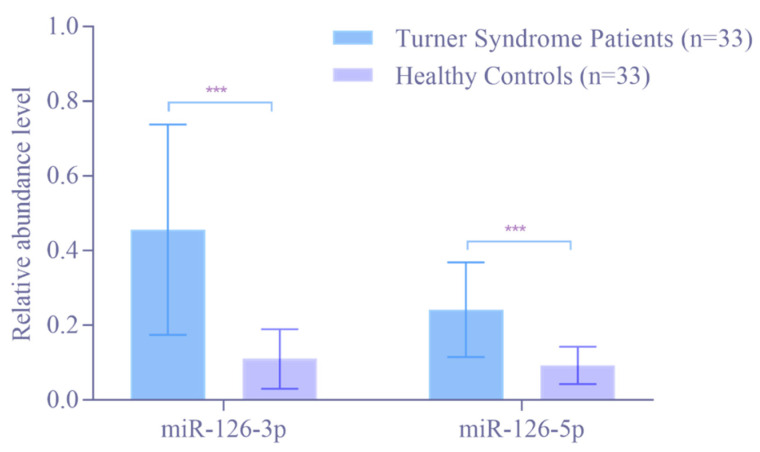
Abundance level of miR-126-3p and miR-126-5p in Turner Syndrome patients compared to healthy volunteers as determined by RT-qPCR. The mean relative abundance level (2^−ΔCt^) of all Turner Syndrome patients and healthy volunteers. RNAU6B was used as an endogenous control for the normalization of miRNA. Unpaired Student’s *t*-test and mean ± standard deviation were used to evaluate differences in abundance levels. *** *p* ≤ 0.001.

To further confirm the higher abundance levels of miR-126-3p and miR-126-5p, their abundance levels were determined in 33 patients with TS and 33 HVs by RT-qPCR analysis. As shown in Figure 1, the abundance levels of both, miR-126-3p and miR-126-5p, were significantly higher in TS patients compared to HVs (*p* < 0.0001). The mean relative abundance level was 5.49-fold higher for miR-126-3p and 2.54-fold higher for miR-126-5p in TS patients compared to HVs.

### 3.4. Correlation between miR-126-3p/5p and Vascular Parameters in Turner Syndrome Patients and Healthy Volunteers

Within the TS cohort, miR-126-3p as well as miR-126-5p correlated significantly with AAO-S and arterial distensibility. Hence, a lower abundance level of miR-126-3p and miR-126-5p was linked with lower values for AAO-S and arterial distensibility and thus a higher aortic stiffness within the TS cohort. In addition, a significant negative correlation was demonstrated within the TS cohort between miR-126-3p and Ea (BSA). Therefore, a lower abundance level of miR-126-3p was linked with a higher value for Ea (BSA) and hence a higher left ventricular afterload within the TS cohort. Moreover, a significant negative correlation was displayed within the TS cohort between miR-126-5p and AAO-D_min_.

Within the HVs, no significant correlations between miR-126-3p, miR-126-5p, and the vascular parameters studied were assessed.

Correlations of miR-126-3p and miR-126-5p with vascular parameters studied are presented for the TS cohort in Table 2.

### 3.5. Diagnostic Accuracy of the miR-126-3p/5p

**Figure 2:**
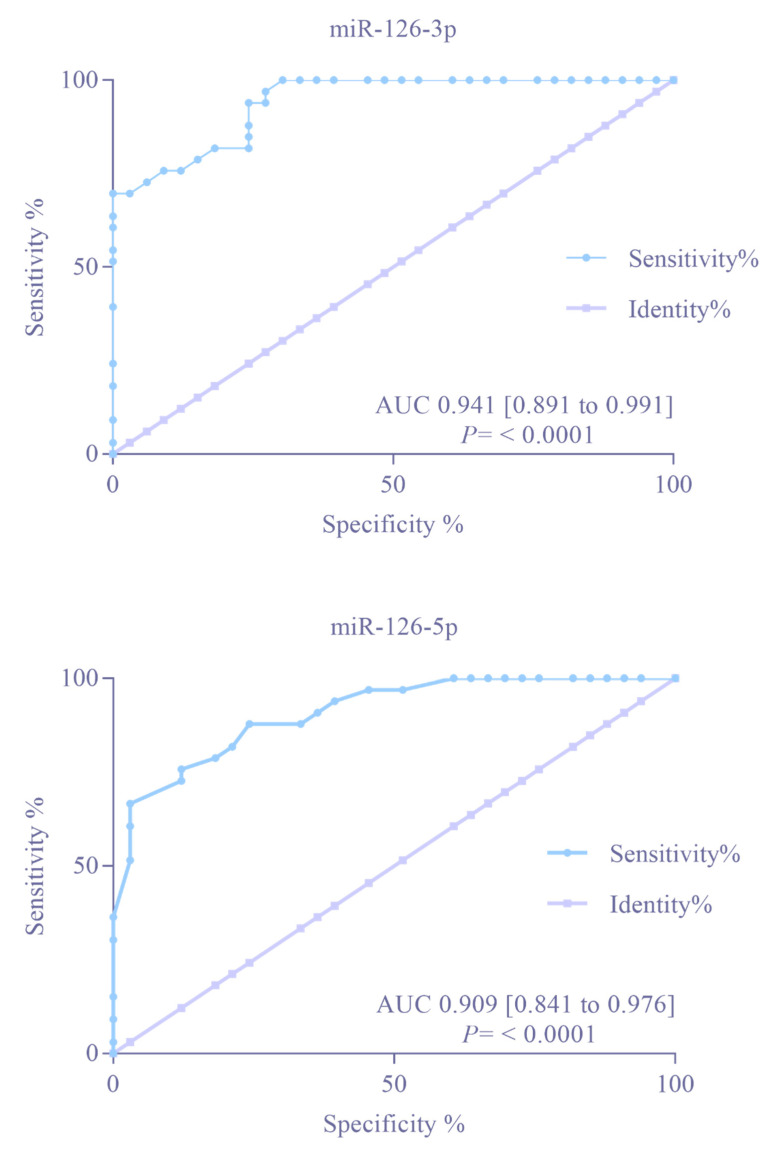
Receiver operating characteristic curves of miR-126-3p and miR-126-5p in Turner Syndrome patients compared to healthy volunteers, as determined by single RT-qPCR.

The AUC value for miR-126-3p and miR-126-5p was computed to evaluate their diagnostic discrimination between TS patients and HVs. Compared with HVs, the AUCs for miR-126-3p and miR-126-5p were 0.941 and 0.909 (*p* < 0.0001), respectively (Figure 2). Together, these AUC values indicate that miR-126-3p and miR-126-5p can discriminate with relatively high accuracy between TS patients and HVs.

## 4. Discussion

This study demonstrated a significantly higher abundance level of miR-126-3p and miR-126-5p in TS patients compared to HVs. Within the TS cohort, the abundance levels of miR-126-3p and miR-126-5p correlated significantly with markers of aortic stiffness (AAO-S and arterial distensibility). In addition, a significant negative correlation between the abundance level of miR-126-3p and left ventricular afterload visualized by Ea (BSA) was shown within the TS cohort. However, no significant correlation was assessed between both miRNAs studied and parameters of peripheral vascular function (e.g., SBP and DBP).

### 4.1. MiR-126-3p/5p and Aortic Stiffness in Turner Syndrome Patients: Pathophysiological Considerations

TS is associated with a general vasculopathy [5,6,7,8]. The results of this study suggest that miR-126-3p and miR-126-5p might be involved in the cardiovascular pathophysiology of TS. Possible pathophysiological considerations will be shared in the following.

Both strands of the pre-miR-126, namely miR-126-3p and miR-126-5p, are highly enriched in endothelial cells and are reported to be involved in the pathophysiology of atherosclerosis [26,31]. An experimental study conducted by Schober et al. demonstrated in mice with genetic deletion of miR-126 pronounced atherosclerotic changes [26,28]. By limiting the expression of vascular cell adhesion molecule 1 (VCAM1), miR-126-3p reduces leukocyte adhesion to endothelial cells and prevents the further pathogenesis of atherosclerosis [32]. The overexpression of miR-126-3p is assumed to have potential anti-inflammatory and anti-atherosclerotic effects [26]. MiR-126-5p is considered to be the even more atheroprotective strand [26,28]. In contrast to miR-126-3p, the inhibition of miR-126-5p was linked with disturbed endothelial repair in mice, underlining its importance of vascular preservation [26,28]. High levels of miR-126-5p suppress Delta-like 1 homolog (Dlk1), a negative regulator of endothelial cell proliferation [28]. In addition, Dlk1 is described to negatively affect angiogenesis by inhibiting NOTCH1 activation [28,33]. Interestingly, shear stress was shown to lower miR-126-5p expression, resulting in reduced endothelial proliferation and cell death and ultimately increased atherosclerosis [26,28]. The upregulation of miR-126-3p and miR-126-5p could potentially be explained as a countermeasure against the general vasculopathy of TS, such as suggested in the literature for hypertensive patients [34]. Within the TS cohort, subjects that displayed a low miR-126-3p abundance level might tend to increased aortic stiffness due to the lower intensity of anti-inflammatory and anti-atherosclerotic effects [26]. In TS patients with pronounced vascular dysfunction, the increased shear stress might lead to lower miR-126-5p expression and hence to lower endothelial proliferation and cell death and increased atherosclerosis, thus aggravating the already present process of arterial stiffening [26]. Consequently, TS patients with a low abundance of miR-126-3p and miR-126-5p might face increased cardiovascular risk.

### 4.2. MiR-126-3p/5p: Potential Biomarkers for Cardiovascular Risk Stratification in Turner Syndrome?

In addition to the results shown in this study, a recent publication of our departments, examining the same TS cohort, was able to demonstrate that the abundance level of miR-126-3p was significantly lower in TS subjects presenting with monocuspid/bicuspid aortic valve disease compared to those with tricuspid aortic valve [17]. In the general population, the presence of a bicuspid aortic valve is linked to endothelial dysfunction and can cause aortic dilatation and dissection later in life [35]. Aortic dissection is one of the major reasons for increased cardiovascular mortality in TS [2]. The risk for aortic dissection in TS is 100-fold higher compared to the general population [10]. Within TS, the presence of a bicuspid aortic valve is associated with a four-fold higher risk for aortic dissection [36]. Hence, these patients are at even greater risk for undesirable cardiovascular events. A study conducted by Kin et al. demonstrated that the expression of miR-126 was significantly downregulated in the plasma of patients with abdominal aortic aneurysm compared to HVs, suggesting its involvement in aortic disease [37]. As a lower miR-126 abundance level was shown to correlate with elevated aortic stiffness and the presence of monocuspid/bicuspid aortic valve, these miRNAs might help in the future as a potential biomarker for early cardiovascular risk stratification in TS. However, validation studies in other TS cohorts are needed to further strengthen the demonstrated results. In addition, quantification assays of miR-126-3p/5p in an independent cohort of patients and age-matched controls are required. Further research with larger sample sizes is necessary to assess the impact of age, karyotype, and cardiovascular morbidity on miR-126-3p and miR-126-5p abundance levels in TS. Moreover, longitudinal studies are needed to evaluate whether miR-126-3p and miR-126-5p might help in the prediction of adverse cardiovascular events in TS.

### 4.3. Limitations

In contrast to a previous publication of our departments [6], no significant differences were demonstrated in vascular function between TS patients and HVs in this study. The relatively small study population undergoing simultaneous genetic and vascular testing can be regarded as one reason for this circumstance. Further, the expression of the miRNAs studied could potentially be altered due to regular medication intake. Interestingly, miR-126-3p and miR-126-5p are assumed to be influenced by estradiol [38,39]. However, the day of the menstrual cycle and estrogen blood levels were not determined in this study. In addition, the endothelial function of study participants was not assessed prospectively. As TS is closely linked to estrogen deficiency, due to ovarian failure, and endothelial dysfunction, further studies are required to evaluate the impact of miR-126-3p/5p on TS health [40,41].

## 5. Conclusions

In this study, the abundance levels of miR-126-3p and miR-126-5p were demonstrated to be significantly higher in TS patients compared to HVs. Within the TS cohort, lower miR-126-3p and miR-126-5p abundance levels were significantly linked with a higher aortic stiffness. This study suggests that miR-126-3p/5p are potentially involved in the cardiovascular pathophysiology of TS. Further studies are required to assess whether miR-126-3p and miR-126-5p might help in the cardiovascular risk stratification of TS patients.

## Figures and Tables

**Table 1:** Vascular function in Turner Syndrome patients (TS) and healthy volunteers (HVs).

Parameters	TS (*n* = 33)	HVs (*n* = 14)	*p*-Value
SBP (mmHg)	121 ± 13.0	117 ± 9.5	0.267
DBP (mmHg)	75 ± 11.9	71 ± 9.8	0.325
PP (mmHg)	46 ± 7.8	46 ± 8.6	0.773
cSBP (mmHg)	109 ± 12.7	104 ± 9.2	0.199
cDBP (mmHg)	77 ± 12.0	73 ± 9.8	0.334
cPP (mmHg)	31 (24–56)	29 (19–46)	0.521
Augmentation Pressure (mmHg)	6.9 ± 3.3	7.3 ± 3.7	0.709
Heart Rate (bpm)	93 ± 11.6	74 ± 13.5	<0.001 ***
AIx@75 (%)	27 (11–61)	24 (8–45)	0.059
PWV (m/s)	4.9 (3.9–6.9)	4.75 (3.9–6.5)	0.321
AAO-D_min_ (cm)	1.3 ± 0.32 ^†^	1.3 ± 0.22	0.992
AAO-D_max_ (cm)	1.4 (1.0–2.2) ^†^	1.6 (1.2–1.8)	0.782
AAO-S (%)	16.2 (4.8–33.3) ^†^	13.8 (6.3–33.3)	0.315
Arterial Distensibility (mmHg^−1^ × 10^−3^)	659 ± 315 ^†^	764 ± 278	0.287
Ea (BSA) (mmHg/mL/m^2^)	3.1 ± 0.83	2.8 ± 0.55	0.272

Mean ± standard deviation is used for normally distributed variables and median (range) for non-normally distributed variables. ^†^ Only 32 TS patients were included; *** *p* ≤ 0.001; SBP, brachial systolic blood pressure; DBP, brachial diastolic blood pressure; PP, brachial pulse pressure; cSBP, central systolic blood pressure; cDBP, central diastolic blood pressure; cPP, central pulse pressure; AIx@75, augmentation index adjusted to a heart rate of 75 bpm; PWV, pulse wave velocity; AAO-D_min_, minimal abdominal aortic diameter; AAO-D_max_, maximal abdominal aortic diameter; AAO-S, abdominal aortic strain; Ea (BSA), arterial elastance indexed to body surface area.

**Table 2:** Correlation of miR-126-3p/5p with vascular parameters in Turner Syndrome patients (TS) (*n* = 33).

Parameters	miR-126-3p (2^−ΔCt^)		miR-126-5p (2^−ΔCt^)	
	r	*p*-Value	r	*p*-Value
SBP (mmHg)	-	ns	-	ns
DBP (mmHg)	-	ns	-	ns
PP (mmHg)	-	ns	-	ns
cSBP (mmHg)	-	ns	-	ns
cDBP (mmHg)	-	ns	-	ns
cPP (mmHg)	-	ns		ns
Augmentation Pressure (mmHg)	-	ns	-	ns
Heart Rate (bpm)	-	ns	-	ns
AIx@75 (%)	-	ns	-	ns
PWV (m/s)	-	ns	-	ns
AAO-D_min_ (cm)	-	ns	−0.43 ^†^	0.0224 *
AAO-D_max_ (cm)	-	ns	-	ns
AAO-S (%)	0.47 ^†^	0.0120 *	0.48 ^†^	0.0090 **
Arterial Distensibility (mmHg^−1^ × 10^−3^)	0.55 ^†^	0.0023 **	0.48 ^†^	0.0095 **
Ea (BSA) (mmHg/mL/m^2^)	−0.48	0.0104 *	-	ns

^†^ Only 32 TS patients were included; * *p* < 0.05; ** *p* ≤ 0.01; SBP, brachial systolic blood pressure; DBP, brachial diastolic blood pressure; PP, brachial pulse pressure; cSBP, central systolic blood pressure; cDBP, central diastolic blood pressure; cPP, central pulse pressure; AIx@75, augmentation index adjusted to a heart rate of 75 bpm; PWV, pulse wave velocity; AAO-D_min_, minimal abdominal aortic diameter; AAO-D_max_, maximal abdominal aortic diameter; AAO-S, abdominal aortic strain; Ea (BSA), arterial elastance indexed to body surface area.

## Data Availability

Data are available from the authors upon reasonable request.
